# Endothelial Dysfunction in the Apolipoprotein E-deficient Mouse: insights into the influence of diet, gender and aging

**DOI:** 10.1186/1476-511X-10-211

**Published:** 2011-11-14

**Authors:** Silvana S Meyrelles, Veronica A Peotta, Thiago MC Pereira, Elisardo C Vasquez

**Affiliations:** 1 Departament of Physiological Sciences, Health Sciences Center, Federal University of Espirito Santo, Vitoria, ES, Brazil; 2 The University of Iowa, Iowa City, IA, USA; 3 Federal Institute of Education, Science and Technology (IFES), Vila Velha, ES, Brazil; 4 Emescam School of Health Sciences, Vitoria, ES, Brazil

**Keywords:** apoE, hypercholesterolemia, atherosclerosis, endothelial dysfunction, oxidative stress, gender

## Abstract

Since the early 1990s, several strains of genetically modified mice have been developed as models for experimental atherosclerosis. Among the available models, the apolipoprotein E-deficient (apoE^-/-^) mouse is of particular relevance because of its propensity to spontaneously develop hypercholesterolemia and atherosclerotic lesions that are similar to those found in humans, even when the mice are fed a chow diet. The main purpose of this review is to highlight the key achievements that have contributed to elucidating the mechanisms pertaining to vascular dysfunction in the apoE^-/- ^mouse. First, we summarize lipoproteins and atherosclerosis phenotypes in the apoE^-/- ^mouse, and then we briefly discuss controversial evidence relative to the influence of gender on the development of atherosclerosis in this murine model. Second, we discuss the main mechanisms underlying the endothelial dysfunction of conducting vessels and resistance vessels and examine how this vascular defect can be influenced by diet, aging and gender in the apoE^-/- ^mouse.

## Background

Because mice are easily bred, have a short generation time and because of the availability of inbred strains, many of which have interesting heritable phenotypes, they are ideal models for the study of genetic contributions to disease [1]. The major disadvantage of the mouse model is its small size, which makes it relatively difficult to perform neural and hemodynamic measurements; however, in our and other laboratories the limitations of the mouse have been overcome through advances in surgical techniques (e.g., vascular catheterization and nerve recording) and in diagnostic imaging methods.

Due to the progressive advancement of molecular biology techniques, it is possible to knockout and restore endogenous genes or to add exogenous genes into the mouse allowing the development of mouse models for human diseases [2-8]. In this brief review, we focus on the apolipoprotein E-deficient (apoE^-/-^) mouse, which is considered the best available model for human lipoprotein disorders and atherosclerosis [9,10]. Specifically, we discuss the main mechanisms underlying endothelial dysfunction in the apoE^-/- ^mouse and examine how this vascular defect can be influenced by diet, aging and gender.

## Lipoproteins and Atherosclerosis Phenotypes

### Murine plasma lipid profiles

The lipid profiles of mice and humans differ greatly. Humans carry approximately 75% of their total plasma cholesterol (PC) on the atherogenic low-density lipoprotein (LDL). Mice naturally carry most of their PC on the antiatherogenic high-density lipoprotein (HDL, ~70 mg/dL) [1,3,11,12]. Normal mice have total PC levels of approximately 80 mg/dL and are highly resistant to atherosclerotic lesions. An exception is the C57BL/6J (C57) mouse strain when it is challenged with a special atherogenic diet [3,13,14]. Interestingly, in this mouse strain, males and females that are fed a diet of normal chow do not differ in their HDL-lipid levels, but females demonstrate a drop (~50%) in their HDL levels when fed an atherogenic diet. Males appear to maintain high HDL levels due to their secretion of testosterone [14]. Thus, for the analysis of lipid metabolism and atherosclerosis in mouse models, diet and gender should be taken into account.

### Plasma lipid profiles and atherosclerosis in the apolipoprotein E-deficient mouse

In 1992, two different groups simultaneously created the first gene-targeted murine model of atherosclerosis by disrupting the antiatherogenic apolipoprotein E (apoE) gene that is involved in cholesterol metabolism [2,3]. The inactivation of the apoE gene is achieved by homologous recombination in mouse embryonic stem cells, usually in a C57 genetic background, to produce apolipoprotein E-deficient (apoE^-/-^) or knockout (apoE KO) mouse. The apoE^-/- ^mouse is the most widely used of the available murine models, and it was the first model to develop vascular lesions that are similar to those observed in humans. ApoE is a glycoprotein with a molecular weight of approximately 34 kDa, which is synthesized in the liver, brain and in macrophages and it is a constituent of chylomicrons and their remnants, very-low-density lipoproteins (VLDL), intermediate-density lipoproteins (IDL) and HDL. ApoE plays a major physiological role in lipoprotein metabolism, and it mediates the high-affinity binding of apoE-containing lipoproteins to the cell-surface LDL receptor and the chylomicron-remnant receptor; therefore, it is an important mediator of the transport and hepatic metabolic clearance of circulating cholesterol [1,3,11,15]. In C57 mice fed a chow diet, values of PC, triglycerides (TG), VLDL+IDL, LDL and HDL levels are approximately 60, 65, 20, 10 and 50 mg/dL, respectively [3,11,16,17]. In the homozygous apoE mutant mouse that is fed a chow diet, there is a shift in plasma lipoproteins from HDL to predominantly VLDL and chylomicron remnant fractions, i.e., most of the PC is in atherogenic lipoprotein fractions. Thus, on a chow diet, apoE^-/- ^mice have increased total PC (~8-fold), TG (1.7-fold), VLDL+IDL (18-fold) and LDL (14-fold), but similar or decreased HDL at all ages [3,11,16-21]. When fed a Western-type diet, a dramatic increase in the proportions of these lipids is observed in total PC (~14-fold) and particularly in the VLDL+IDL lipoprotein fraction (~30-fold) [3,19,20,22-24]. When fed a normal diet apoE^-/- ^mice exhibit monocyte attachment to endothelial cells and a disruption of the subendothelial elastic lamina at 6-8 weeks of age, lesions containing foam cells and smooth muscle cells are seen at 8-10 weeks of age, and fibrous plaques appear at 15-20 weeks of age [3,25]. At ~70 weeks-old, apoE^-/- ^mice exhibit over 90% occlusion of the aortic lumen, and a similar percentage of occlusion is observed at 32 weeks when the animals are fed a Western-type diet [26]. Sites of predilection include the aortic root, aortic arch, common carotid, superior mesenteric, renal and pulmonary arteries [25,27]. Thus, similar to humans, apoE^-/- ^mice display increased levels of total PC and extensive lipid deposition in the major large arteries, which is accelerated and aggravated when they are fed a Western-type diet, suggesting a similarity with humans.

### The influence of gender on atherosclerosis

**Figure 1:**
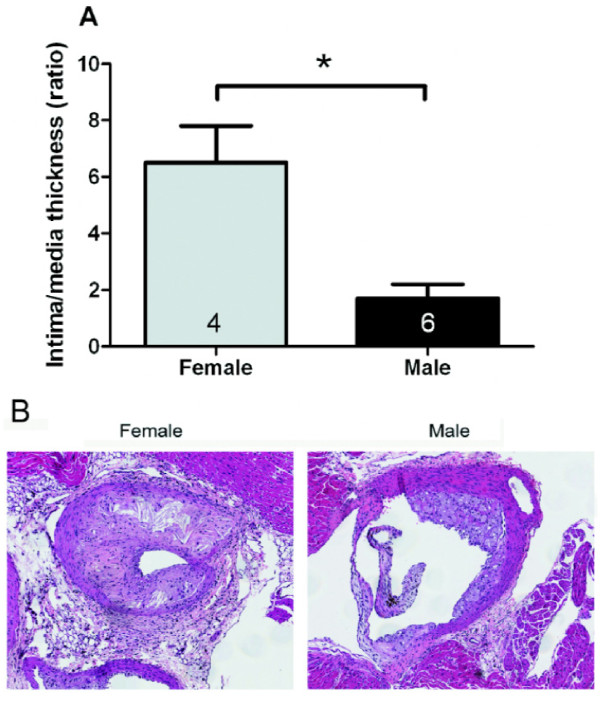
**Effect of gender on atherosclerotic lesions in the aortic root of 8- to 10-week-old apoE^-/- ^mice fed for 3 months with a high-fat diet**. Average aorta thickness is significantly higher in females than males (A). Hematoxylin-eosin stained cross sections at the root of the aorta show remarkable differences in the atherosclerotic lesion between the female and the male mouse (B). Reproduced from Smith et al. [24] with permission.

**Figure 2:**
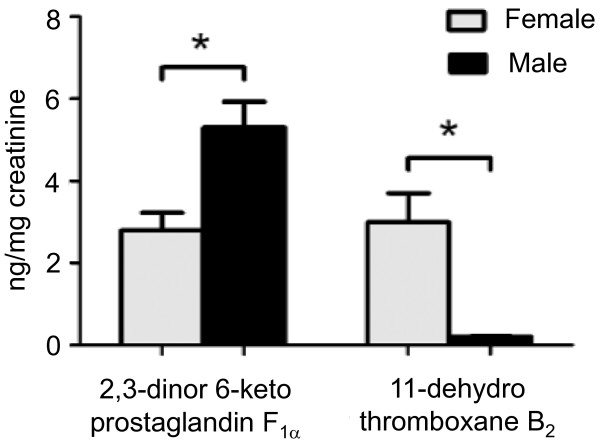
**Effect of gender on the levels of 2,3-dinor-6-keto prostaglandin F_1α _and 11-dehydro thromboxane B_2 _in 24-hour urine samples of ApoE^-/- ^female and male mice after a 3-month high fat diet regimen**. The data presented are as mean ± SEM. * p < 0.05, when compared to males. Reproduced from Smith et al. [24] with permission.

The influence of gender on atherosclerosis and the protective effect of estrogen are controversial. The majority of studies show that during their reproductive years, women are less prone to developing cardiovascular diseases and atherosclerosis than men, but men and post-menopausal women at comparable ages are at an equal risk for developing the disease as, as recently reviewed [28]. Consequently, cardiovascular diseases in women develop, on average, 10 years later than they do in men. In mice, large number of studies using apoE^-/- ^or LDL receptor-knockout mice have demonstrated an atheroprotective role for endogenous estrogen and estrogen replacement in females (reviewed 5 years ago [29]). However, after that time, emerged convincing evidence suggests that atherosclerotic lesions in apoE^-/- ^mice are greater in females than in males, and the reasons for this discrepancy are not clear. Therefore, the influence of gender on atherosclerosis in the apoE^-/- ^mouse is still under debate. For example, Tangirala et al. [30] showed that in LDL receptor-deficient mice, the incidence of atherosclerotic lesions in the aorta was significantly higher in males than in females. However, in apoE^-/- ^mice (mixed C57 × 129ola genetic background) fed a Western-type diet for 6 months (starting at 5-6 months old), they observed only a trend towards increased lesions in males compared to females (not significant). Similarly, Coleman et al. [26] did not encounter clear-cut gender differences in the histopathology of aortic arch atherosclerosis in 6- to 80-week-old apoE^-/- ^mice (C57 genetic background) fed a standard chow diet. In addition, Elhage et al. [31] showed that although the total PC was higher in males than in females in apoE^-/- ^mice (C57 × 129/B6 genetic background) fed a chow diet, there was no statistical significance in the fatty streak areas between genders. In contrast, a recent study by Chiba et al. [32] showed that in apoE^-/- ^mice (C57 genetic background) fed an atherogenic diet for 16 weeks, starting at 10 weeks of age, there were significant less atherosclerotic lesions in females than in males and greater in ovariectomized than in sham animals, without differences in serum lipoproteins between genders. The protective influence of female hormones against atherosclerosis in the apoE^-/- ^mouse is corroborated by the findings that estrogen administered to apoE^-/- ^mice (C57 × 129ola genetic background) prevented fatty streak formation in female and male mice fed a chow diet [31] and reduced atherosclerotic lesion development in mice fed a Western-type diet [33]. In contrast, there is a growing body of convincing evidence against the atheroprotective effects of estrogen. For example, tamoxifen, which exhibits tissue-specific estrogen receptor agonist/antagonist activities and has been shown to act as a cardioprotective agent in postmenopausal women [34], decreased cholesterol levels by 7-fold and abolished lipid lesion development in apoE^-/- ^mice that received this therapy repeatedly [35]. In agreement with these observations, Caligiuri et al. [36] observed that in apoE^-/- ^mice (C57 genetic background) fed a normal chow diet, young female mice developed larger and more advanced atherosclerotic lesions compared with young male mice. The data from this latter study suggest that differences in atherosclerotic lesions between genders may be related to differences in the cellular immune responses to the atherosclerotic-related autoantigen, oxidized LDL (oxLDL), in the different sexes [36]. The presence of apoA in the HDL may also be a sex-related determinant for receptor interactions and this may be of pathophysiological importance in atherosclerosis. Indeed, compared to male apoE^-/- ^mice, female apoE^-/- ^mice have lower plasma levels of apoA-I and apoA-II [21]. Moreover, it has been reported that in female mice, HDL and apoA-I are negatively associated with aortic atherosclerotic lesions, while the association with apoA-II was positive. In contrast, in males no significant associations were observed [21], indicating that changes in HDL and apoA are important determinants of atherosclerosis in females but not in males. Accordingly, in apoE^-/- ^mice, fed a standard chow diet [21,36] or a Western-type diet [5,24], females exhibit areas of larger vascular atherosclerotic lesions than males (see Figure 1). In a study it was also demonstrated that the elevated production of thromboxane (TXA_2_) and the reduced production of prostacyclin (PGI_2_) observed in female apoE^-/- ^mice (Figure 2) are gender-related proatherogenic risk factors in these animals [24]. Caligiuri et al. [36] showed that vascular atherosclerotic lesions were larger and more advanced in young (16-week-old) female apoE^-/- ^mice compared to male apoE^-/- ^mice, but in aged (48-week-old) animals, when the blood levels of estrogen decreased in females, there was no longer a sex difference in lesion size [36]. These data suggest that age- and sex-dependent variations of cell-mediated immune responses modulate the onset and progression of atherosclerosis. In support of these findings, a recent study showed that in apoE^-/- ^mice, atherosclerosis was reduced following ovariectomy and was aggravated following treatment with 17-β-estradiol at doses that were similar to physiological levels of the hormone [37]. In view of these findings, the apoE^-/- ^mouse model allows the investigation of the detrimental effects of 17-β-estradiol on atherosclerosis and contributes to clinical studies that reveal the unfavorable effects of hormone replacement therapy in postmenopausal women. Although the influence of female gender on atherosclerosis is still controversial and more research is needed to understand fully the role played by estrogens in atherosclerotic lesion in the apoE^-/- ^mouse, there is convincing evidence that at a young age and on a normal chow diet, females develop greater atherosclerotic lesions than males. Indeed, the above study reporting greater atherosclerotic lesions in males than in females, the animals were 26-week-old and fed an atherogenic diet [32], whereas in the study reporting the opposite observation, the animals were younger and fed a regular chow diet and significant differences between the genders in the older animals were not observed [36]. In agreement with these observations, Maeda et al. [38] showed that atherosclerotic lesions at the aortic roots of apoE^-/- ^mice (C57 genetic background) fed a regular chow diet developed plaques earlier in the females than in the males. Taken together, the above studies highlight the importance of considering sex in the analysis of atherosclerosis and lipid metabolism in the apoE^-/- ^mouse model, and they fuel the debate on the effects of estrogen on atherosclerosis in murine models.

Therefore, the deletion of the single apoE gene, which results in severe hypercholesterolemia, is sufficient to convert the mouse from a species that is highly resistant to atherosclerosis to one that is highly susceptible to the disease [2,3,11]. The characterization of the morphology, histology, pathology and mechanisms (but not gender) involved in the development of vascular atherosclerotic lesions in this murine model of atherosclerosis has been broadly studied for two decades and has been previously reviewed [1,9,10]. However, the vascular endothelial dysfunction in the apoE^-/- ^mouse has not been fully elucidated, and the main purpose of this brief review is to summarize and discuss the mechanisms underlying this vascular defect and to address the influence of diet, aging and gender.

## Endothelial Function in Physiological Conditions

### Assessment of endothelial function and the role of nitric oxide (NO)

**Figure 3:**
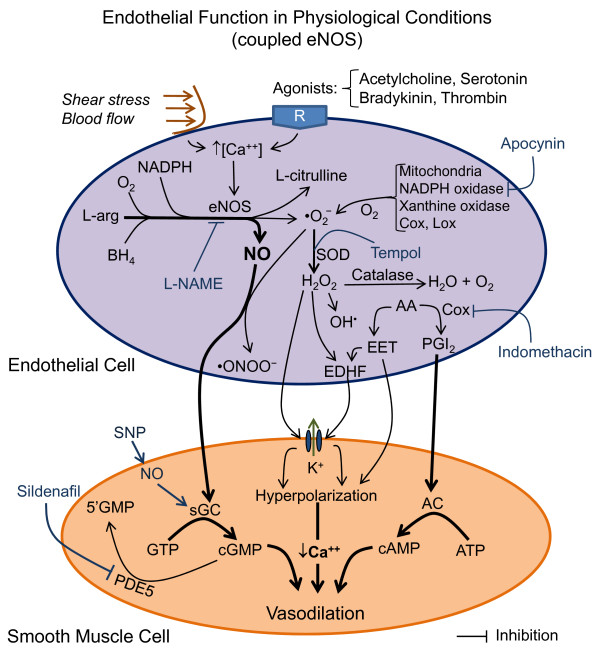
**Hypothetical scheme illustrating the possible biochemical pathways for the production of nitric oxide (NO) and reactive oxygen species (ROS) by endothelial cells under physiological conditions, including specific pharmacological tools that are used to study the vascular function (blue lines)**. The predominant pathways that lead to normal endothelial vasodilation are indicated by thick lines/arrows. Abbreviations: AA, arachidonic acid; AC, adenylate cyclase; BH_4_, tetrahydrobiopterin; cAMP, 3'-5'-cyclic adenosine monophosphate; cGMP, cyclic guanosine monophosphate; Cox, cyclooxygenases; EDHF, endothelium-derived hyperpolarizing factor; EET, epoxyeicosatrienoic acids; eNOS, endothelial nitric oxide synthase; H_2_O_2_, hydrogen peroxide; L-arg, L-arginine; L-NAME, L-nitroarginine methylester; Lox, lipoxygenases; NADPH, nicotinamide adenine dinucleotide phosphate; •O_2_^-^, superoxide anion; •ONOO^-^, peroxynitrite; PDE5, phosphodiesterase type 5; PGI_2_, prostacyclin; sGC, soluble guanylyl cyclase; SOD, superoxide dismutase; SNP, sodium nitroprusside.

The general aspects of endothelial function and atherosclerosis have been reviewed previously [39-41], and are summarized here. The vascular endothelium regulates vascular tone and structure in conductance and resistance vessels through a continuous release of a variety of autocrine and paracrine vasoactive mediators such as nitric oxide (NO), reactive oxygen species (ROS), endothelin-1 (ET-1), angiotensin (Ang) II, endothelium-derived hyperpolarizing factor (EDHF), prostacyclin (PGI_2_) and epoxyeicosatrienoic acids (EET). Importantly, the position of the endothelium in the vessel wall makes it a primary target for injuries. As shown in the schematic representation in Figure 3, shear stress and acetylcholine (ACh), which is commonly used to asses endothelial function, and receptor-specific agonists, such as bradykinin, thrombin and serotonin, causes increase in intracellular Ca^++ ^levels. This increase in Ca^++ ^activates endothelial NO synthase (eNOS), which acts on L-arginine (L-arg) resulting in the production of NO. This reaction requires the participation of cofactors, such as tetrahydrobiopterin (BH_4_) and NADPH, which are critical for coupling the reduction of molecular oxygen (O_2_) and the oxidation of L-arg, resulting in the production of NO and L-citrulline [42-45]. NO diffuses to the underlying vascular smooth muscle cells (VSMC), where it activates soluble guanylyl cyclase (sGC) to produce cyclic guanosine monophosphate (cGMP) from guanosine triphosphate (GTP), and induces a decrease in Ca^++ ^followed by VSMC relaxation.

### Endothelial generation of reactive oxygen species (ROS) and other vasoactive mediators

As illustrated in Figure 3, this reaction of the endothelial cell involving the L-arg-eNOS system also generates ROS, including the free radicals (unpaired electrons) superoxide anions (•O_2_^-^), peroxynitrite (•ONOO^-^), and hydroxyl radicals (OH^•O^) and non-radicals, such as hydrogen peroxide (H_2_O_2_), that are involved in diverse cardiovascular diseases [46-50]. Briefly, •O_2_^- ^reacts extremely rapid with NO generating •ONOO^-^. Superoxide anions are also rapidly scavenged by the antioxidant enzyme SOD, thus protecting NO and generating H_2_O_2_. Under physiological conditions, this interaction is minimized by endogenous antioxidant defenses, such as SOD, and the low levels of ROS act as signals to modulate proliferation, apoptosis and gene expression through the activation of transcription factors. ROSs are produced by all vascular cell types, and have been implicated in the regulation of vascular tone by modulating vasodilation directly or indirectly by decreasing NO bioavailability through quenching by •O_2_^- ^to form •ONOO^-^, which is a short-lived species that impairs endothelial function. In contrast, high concentrations and/or the inadequate removal of ROS, especially •O_2_^-^, results in oxidative stress, which has been implicated in the pathogenesis of many cardiovascular diseases including hypercholesterolemia and atherosclerosis.

Endothelium releases other potent vasodilator substances, such as PGI_2_, an eicosanoid of the cyclooxygenases pathway. The activities of PGI_2 _include the activation of adenylate cyclase (AC) in VSMCs, which results in the generation of 3'-5'-cyclic adenosine monophosphate (cAMP), causing a relaxation of the VSMC in most blood vessels. An additional relaxant pathway acts through the release of EDHF that diffuses to and activates VSMC potassium channels, causing hyperpolarization [47]. Reliable data [47-49], indicate that H_2_O_2 _derived from the dismutation of •O_2_^- ^may act as an EDHF because it elicits hyperpolarization and vasodilation by activating VSMC potassium channels. In large vessels, H_2_O_2_-induced relaxation can be endothelium-independent and endothelium-dependent, as indicated by the observation that the eNOS inhibitor L-nitroarginine methylester (L-NAME) abolishes this effect [51,52]. The vasodilator activity of H_2_O_2 _also affects mesenteric and coronary arteries, and in coronary arteries, it has been shown to involve COX-1 and the VSMC potassium channel [53,54]. Therefore, there are substantial data indicating that the four main mediators of endothelial vasodilator function, NO, H_2_O_2_, PGI_2 _and EDHF, interact in a coordinated manner to maintain normal endothelial function [55,56]. In terms of relative importance, studies [51,52,54] show that NO is the predominant endothelium-derived vasodilator in large arteries, whereas in resistance vessels of the microcirculation EDHF predominates over other agents. The impairment of vascular function, which is associated with several cardiovascular events and atherosclerosis, has typically been characterized by one or more of the following responses: decreased endothelium-dependent vascular relaxations, decreased endothelium-independent vascular relaxations, and/or increased vasoconstrictions [57-60].

## Endothelial Function and Dysfunction in the ApoE^-/- ^Mouse

### The influence of diet, age and gender on endothelial function of large arteries

**Figure 4:**
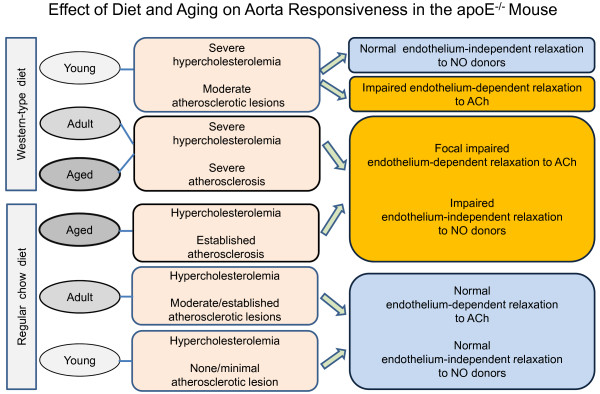
**Diagram showing the effect of diet and aging on aortic endothelium-dependent relaxation in response to acetylcholine (ACh) and endothelium-independent relaxation in response to nitric oxide (NO) donors in apoE^-/- ^mice, based on the majority of studies reviewed**.

One hallmark of atherosclerosis is vascular dysfunction, which is observed mainly in large vessels and has generally been defined by a decreased in endothelium-dependent vasodilation that is generally considered to precede the development of atherosclerosis and to predispose humans to the development of structural vascular changes [61]. However, this general concept is not fully supported by studies in the murine model of spontaneous hypercholesterolemia and atherosclerosis. Indeed, aortic rings isolated from young (6-18-week-old) male and female apoE^-/- ^mice fed a standard chow diet (hypercholesterolemia only) exhibit a preserved endothelial NO-dependent relaxation response to ACh when compared with wild-type control mice [12,62,63]. Similar results have been observed in aortas from adult (20-35-week-old) male [64] and female [4,65] apoE^-/- ^mice. In contrast, in aged apoE^-/- ^mice (50-70-week-old) that exhibit both hypercholesterolemia and established atherosclerosis, an endothelial dysfunction, as demonstrated by a significantly blunted aorta relaxation response to ACh, has been observed [62,66,67]. When apoE^-/- ^mice are fed a Western-type diet to accelerate and aggravate hypercholesterolemia and atherosclerosis, the vasodilation response to ACh in the aortas [23,64,65] and carotid arteries [23] of ~20-30-week-old males is normal; however, studies have shown a significant impairment of the vasodilation response to ACh in 14-15-week-old male [22,65,68-76] and female [4,19,77] mice. Crauwels et al. [66] demonstrated that in aged (72-week-old) apoE^-/- ^mice, the aortic endothelial dysfunction is a focal and not a systemic hypercholesterolemia-dependent defect, i.e., it is strictly associated with plaque formation. Overall, the above findings (see diagram in Figure 4) suggest that endothelial function in the aortas of apoE^-/- ^mice is normal at the early stages of the pathology and the impairment of endothelial NO-mediated dilation occurs at a later stage, mainly in aged animals and when mice are fed an atherogenic Western-type diet, which aggravates hypercholesterolemia and atherosclerosis.

Recent evidence suggests that gender plays an important role in endothelial dysfunction in the large vessels of apoE^-/- ^mice. Indeed, atherosclerosis in apoE^-/- ^mice was reduced and the endothelial dysfunction of aortic rings was attenuated following ovariectomy and was aggravated by treatment with 17-β-estradiol at doses that were near physiological levels [37]. Hence, the apoE^-/- ^mouse, in addition to being a model for human atherosclerosis, appears to be a suitable experimental model for studying the detrimental effects of 17-β-estradiol on endothelial dysfunction [37]. Despite a growing body of evidence suggesting that gender influences atherosclerosis in apoE^-/- ^mice [30-38], few studies have investigated the differences in endothelial function between males and females. More interesting is that a large number of publications have not revealed the gender of the mice used in the studies.

**Figure 5:**
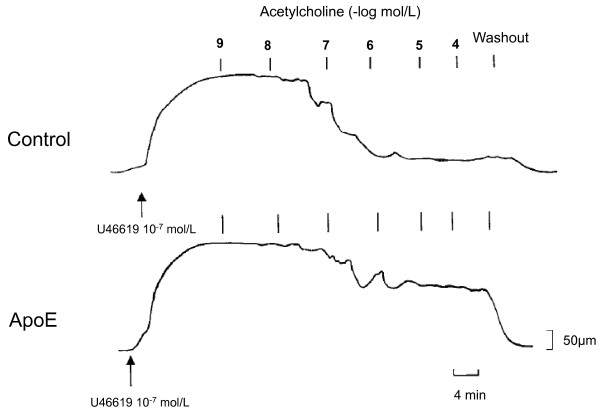
**A tracing illustrating the reduced acetylcholine-induced vasorelaxation of a common carotid artery ring that was precontracted using the thromboxane analog U46619 in a male apolipoprotein E-deficient (ApoE) mouse compared with a wild-type C57BL/6J (control) mouse**. Both animals were fed a lipid-rich Western-type diet for 26 weeks. Reproduced with permission from d'Uscio et al. [80].

In humans, there is a general concept that endothelial dysfunction precedes the development of atherosclerosis [41]. Moreover, there is evidence that the impaired endothelial NO-dependent relaxation response to ACh in apoE^-/- ^mice is not determined by hypercholesterolemia alone. The hypothesis that endothelial dysfunction in large vessels of apoE^-/- ^mice is dependent on plaque formation was tested by Bonthu et al. [12] using a different experimental design. They examined the endothelium-dependent relaxation of aortic rings from 19-week-old apoE^-/- ^mice and apoE^-/-^/LDL receptor-deficient (double knockout) mice fed a standard chow diet and compared them with wild-type C57mice. Relaxation in response to ACh of the proximal and distal segments of thoracic aortas from apoE^-/- ^mice (atherosclerotic lesions were minimal or absent) was normal; however, the relaxation response was greatly impaired in the proximal segments of thoracic aortas (containing atherosclerotic lesions) from the double knockout mice but not in distal segments that had minimal or no atherosclerotic lesions. Similarly, others have observed a decrease in NO-dependent vasodilation in atherosclerosis-prone regions, whereas it was preserved in regions that are less prone to atherosclerosis in the descending thoracic aorta of female apoE^-/- ^mice fed a high-cholesterol diet [77]. These findings indicate that aortic endothelial dysfunction in mice is a focal (plaque-related) and not a systemic hypercholesterolemia-dependent defect [66]. Supporting this observation, is the report that human apoAI transgenesis, which is known to raise HDL, attenuates atherogenesis and improves aortic vasomotor responses to ACh in apoE^-/- ^mice [4] in vessel segments that exhibit large lesions but not those with smaller lesions [66]. This observation explains the association of high plasma HDL levels with the normal endothelium-dependent vasorelaxation that is observed in humans [78]. However, this conclusion may have limitations when considering conductance arteries other than the aorta. For example, young (~15 weeks old) [79] or adult (~30 weeks old) [80] apoE^-/- ^mice demonstrate impaired endothelial-dependent relaxation in the common carotid arteries despite the fact that no morphological changes were observed in these vessels at that age (illustrated in Figure 5). These observations suggest that in contrast to the aorta, endothelial dysfunction can occur in other non-atherosclerotic arteries and that the apoE^-/- ^mouse carotid artery is a valuable experimental model for endothelial dysfunction in conditions of hypercholesterolemia alone.

### The role of endothelial NO bioavailability and endothelin in endothelial dysfunction of large arteries

**Figure 6:**
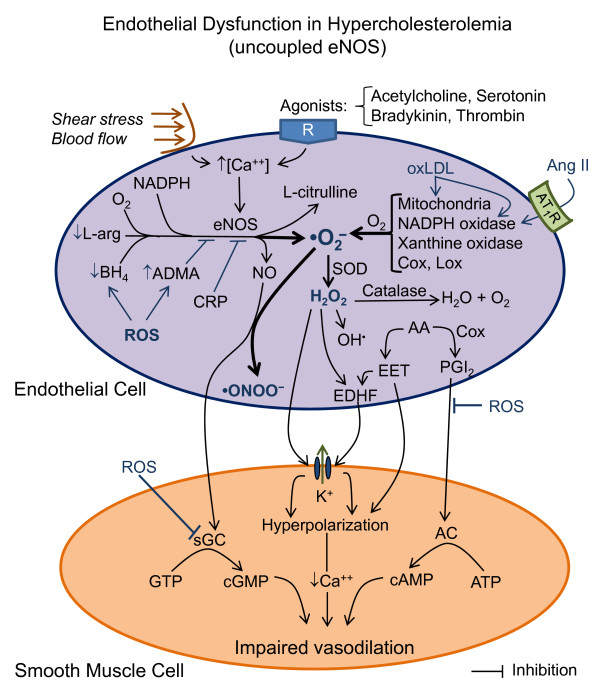
**Hypothetical scheme illustrating the possible mechanisms that lead to endothelial nitric oxide synthase (eNOS) uncoupling (thick lines/arrows), which results in a reduction of nitric oxide (NO) release and an exacerbation of superoxide anion (•O_2_^-^) production, generating peroxynitrite (•ONOO^-^) and thus, leading to impaired vasodilation**. The main causes of the generation of excessive •O_2_^- ^and the resultant endothelial dysfunction are highlighted. Abbreviations (some are listed in Figure 3): ADMA, asymmetric dimethyl-L-arginine; Ang II, angiotensin II; AT_1_R, Ang II type 1 receptor; CRP, C-reactive protein; Lox, lipoxygenases; oxLDL, oxidized low-density lipoprotein.

The endothelial dysfunction of large vessels in hypercholesterolemia and other cardiovascular diseases has been attributed to the following: (a) a decrease in NO production or eNOS synthesis/activity; (b) excessive production of vascular ROS, where •O_2_^- ^reacts with NO, resulting in the formation of •ONOO^- ^and a decreased in the bioavailability of NO; (c) the local oxidation of circulating lipoproteins and/or (d) a decreased antioxidant capacity (see scheme in Figure 6). There is evidence that the chemical inactivation and reduced biosynthesis of NO are the key mechanisms responsible for endothelial dysfunction in the aortas of atherosclerotic apoE^-/- ^mice [12,69]. Vascular relaxation responses to ACh are attenuated by the inhibition of NOS by L-NNA in aortas from normal mice and in segments of aortas that have no intimal thickening in apoE^-/- ^mice [12]. Similarly, aortas from young apoE^-/- ^mice fed a regular chow diet exhibit normal eNOS expression and normal dilation responses to ACh [63,64]. However, transfer of the eNOS gene into apoE^-/- ^mice carotid arteries *in vivo *results in increased eNOS expression levels and normalized relaxation responses to ACh [72]. Interestingly, apoE^-/- ^mice fed a Western-type diet exhibit increased vascular ET-1 production, reduced endothelial NO release, and impaired endothelium-dependent relaxation, which are all normalized by chronic blockage of the ET_A _receptor [68]. These data suggest that a key mechanism underlying the endothelial dysfunction in atherosclerotic large vessels in apoE^-/- ^mice is a decreasing bioavailability of NO, which appears to be associated with the activation of the ET-1 system. From a clinical viewpoint, the current data demonstrate that the blockage of the ET_A _receptor may have therapeutic potential in patients suffering from endothelial dysfunction [81].

### The role of reactive oxygen species (ROS) and angiotensin in endothelial dysfunction of large arteries

Reduced endothelial NO bioavailability has also been attributed to a deficiency in substrates or cofactors or to the enhanced formation of •O_2_^-^, which reacts with NO in apoE^-/- ^mice [7,69] and in other experimental and clinical studies of cardiovascular pathophysiologies [58,59]. In hypercholesterolemia, when lipids can be incorporated into the endothelium, eNOS becomes uncoupled leading to the generation of ROS (including •O_2_^-^, H_2_O_2_, OH^•O^, and the strong oxidant •ONOO^-^) instead of NO. Taking into consideration that BH_4 _is highly susceptible to oxidative degradation by •O_2_^- ^or •ONOO^-^, the initial degradation of BH_4 _by ROS derived from another source induces eNOS uncoupling and the amplification of oxidative stress in conditions of hypercholesterolemia and atherosclerosis in human [82] and in apoE^-/- ^mice [83]. Indeed, endothelial dysfunction is abolished by BH_4 _supplementation in hypercholesterolemic patients [82]. Thus, depletion of BH_4 _(e.g., oxidized by •ONOO^-^) may have an impact on turning eNOS into an •O_2_^- ^generating enzyme and thereby may lead to endothelial dysfunction. In apoE^-/- ^mice endothelial dysfunction is associated with increased •O_2_^- ^and decreased eNOS activity [7,69]. NADPH oxidase, which transfers electrons from NADH or NADPH to molecular oxygen to produce •O_2_^-^, is necessary for the normal progression of atherogenic lesions in apoE^-/- ^mice [84,85]. In the conducting vessels of hypercholesterolemic mice, it has been reported that NADPH oxidase contributes markedly to the impairment of endothelium-dependent vasodilatation by inactivating NO and increasing oxidative stress [85,86]. Accordingly, the inhibition of NADPH oxidase by apocynin improves ACh-induced relaxation in the superior mesenteric artery [86]. Similarly, an SOD mimetic was shown to reverse aortic endothelial dysfunction in apoE^-/- ^mice by decreasing the levels of NADPH oxidase-dependent •O_2_^- ^[87]. In support of these findings, it was shown that antianginal and anti-ischemic ivabradine reduced vascular NADPH oxidase activity, improved aorta endothelial function and reduced atherosclerotic plaque in young apoE^-/- ^mice fed a Western-type diet [74]. Moreover, apoE^-/- ^mice that are genetically deficient in NADPH oxidase show retarded development of atherosclerosis in the aorta [84]. Angiotensin also plays an important role in this process, as demonstrated by Daugherty et al. [88] in LDL receptor-deficient and Ang AT_1A _receptor-deficient mice. In addition to the augmented systemic renin-angiotensin system, locally formed Ang II appears to play an important role in the mechanism that affects NO bioavailability, as indicated by the discovery that hypercholesterolemia may trigger the upregulation of vascular chymase, which may be involved in intimal lipid deposition, and may facilitate the development of atherosclerosis [89]. In apoE^-/- ^mice, Ang II also contributes to increased NADPH-dependent vascular •O_2_^- ^production and is implicated in the pathogenesis of atherosclerosis and endothelial dysfunction in this model [7,90-93]. This concept is supported by the finding that the inhibition of Ang II normalizes vascular •O_2_^- ^and NADPH oxidase activity and improves endothelial dysfunction in young atherosclerotic animals [94]. This observation suggests a crucial role for Ang II-mediated •O_2_^- ^production in the early stages of atherosclerosis. Contrarily, some fragments of the renin angiotensin system can counteract the deleterious actions of ROS, as indicated by the finding that the treatment of apoE^-/- ^mice with Ang IV [75] or Ang 1-7 [76] improved aortic endothelial function, which was associated with decreased •O_2_^- ^levels and increased eNOS expression, i.e. increased NO bioavailability [75,76]. Therefore, the uncoupling of eNOS in the endothelium may lead to oxidative stress and endothelial dysfunction via (a) the diminished enzymatic production of NO, (b) the increased production of •O_2_^- ^contributing to oxidative stress, and (c) the simultaneous production of NO and •O_2_^-^, generating •ONOO^-^.

### The role of endothelial antioxidant enzymes and smooth muscle cell cGMP and cAMP

Impairment of endothelial dysfunction in large vessels may also be related to the abnormal degradation and/or inactivation of NO by •O_2_^- ^because of the incorporation of lipids within the endothelium [12]. Thus, the functional integrity of antioxidant enzymes e.g., SOD, glutathione peroxidase, heme and oxygenase, is important for protection against oxidative stress and endothelial dysfunction. Accordingly, aortas from apoE^-/- ^mice fed a Western-type diet showed an impaired dilation response to ACh and a decreased SOD activity compared to apoE^-/- ^mice fed a normal chow diet [65]. Although reports show that SOD protein expression is unaltered in aorta that are exposed to hypercholesterolemia [69], SOD mimetics reduced •O_2_^- ^production and partially normalized relaxation in response to ACh in aortic and carotid arteries from apoE^-/- ^mice [69,80,87]. Moreover, increased •O_2_^- ^levels and impaired relaxation in response to ACh were observed in the carotid arteries of aged (~65-week-old) apoE^-/- ^mice that are heterozygous for the mitochondrial isoform of SOD2 (apoE^-/-^/SOD2^+/-^) compared with mice that are homozygous for the gene (apoE^-/-^/SOD2^+/+^), although PC levels and intimal areas were similar [8]. Thus, during the last three decades, accumulating evidence suggests that the main mechanism involved in endothelial dysfunction in hypercholesterolemic mice is the decrease in the bioavailability of endothelial NO due to a reduction in eNOS activity and/or a breakdown of NO by •O_2_^-^. Because of these new insights into the mechanisms of endothelial dysfunction, it is anticipated that specific therapies will be developed to prevent and treat the endothelial dysfunction that is observed in hypercholesterolemia and atherosclerosis.

Some studies have examined the hypothesis that the vascular dysfunction may be dependent not only on the reduced bioavailability of NO but also on the altered responsiveness of VSMCs to NO. Therefore, the functional integrity of the VSMC has been tested to determine the contribution of this vascular layer to the vascular dysfunction. Interestingly, cGMP levels were significantly reduced in the atherosclerotic aortas [69] but not in the carotid arteries without morphological changes [80] from male apoE^-/- ^mice fed a Western-type diet, indicating that a selective loss of cGMP-dependent vascular function is associated with atherosclerosis in this animal model. Others have shown that in apoE^-/- ^mice exhibiting normal [4,12,16] or impaired [4,6,7,62,71,73,80,85] endothelial NO-dependent relaxations, the endothelium-independent vasorelaxation in response to an NO donor, i.e., sodium nitroprusside (SNP), was not affected, indicating that the responsiveness of the VSMC to NO is preserved. However, an attenuated endothelium-independent response was observed in atherosclerotic aortas from apoE^-/- ^mice fed a lipid-rich Western-type diet [69] but it was not observed in non-atherosclerotic carotid arteries [80]. Thus, despite some controversies, there is evidence that the apoE^-/- ^mouse, in addition to its endothelial dysfunction, exhibits decreased VSMC responsiveness to NO in conducting arteries that have atherosclerotic lesions but not in conducting arteries that do not have atherosclerotic lesions.

### Promising approaches for the treatment of vascular wall oxidative stress and endothelial dysfunction

The incorporation of lipids within the endothelium, an early manifestation of atherosclerosis, and the associated oxidative processes may contribute to the degradation of NO [12]. The assumption that oxLDL plays a pivotal role in the pathogenesis of endothelial NO dysfunction is based on the findings of Jiang et al. [22], who demonstrated that the *in vitro *treatment of aorta with oxLDL mimicked the endothelial NO dysfunction that was observed in apoE^-/- ^mice. Moreover, adenovirus-mediated gene transfer of the human paraoxonase 1 (PON1), an HDL-associated enzyme that destroys lipid peroxides, into aged apoE^-/- ^mice with advanced atherosclerosis, decreased the oxLDL content of the plaques and restored endothelial function in plaque-bearing but not in plaque-free segments of the thoracic aorta [67]. In addition, macro and microvascular function in apoE^-/- ^mice was restored by treatment with large empty phospholipid vesicles, which accelerates the reverse pathway of lipid transport from peripheral tissues to the liver [95]. These data show that oxLDL plays an important role in the pathogenesis of endothelial NO dysfunction in apoE mice and points to additional promising approaches for the treatment of vascular wall oxidative stress and endothelial dysfunction in atherogenic hyperlipidemia. As reviewed elsewhere [39,60], several studies of the therapeutic effect of lipid-lowering statins on endothelial dysfunction have resulted in an improvement of the endothelium-dependent dilation of coronary and peripheral arteries in patients, independent of its PC-lowering effects. In hypercholesterolemic apoE^-/- ^mice, it has been shown that statin treatment promotes eNOS function in aortic extracts [96]. In apoE^-/- ^mice suffering from hypercholesterolemia and atherosclerosis, statin therapy prevents the deficit in the bioavailability of NO in carotid arteries [23]. Interestingly, statins prevent the enhanced vasoconstrictor response to ET-1 of aortas in apoE^-/- ^mice, independent of their lipid-lowering properties [97]. The potential of statins for the prevention and treatment of endothelial dysfunction in the apoE^-/- ^mouse is currently under intense investigation. Other therapies have also been tested, including the systemic administration of rapamycin-eluting stents, which have been used for percutaneous coronary interventions and are associated with high eNOS and protection against atherosclerosis. Although rapamycin treatment can protect against atherosclerosis in carotid arteries [98], studies from our laboratory show that this agent does not affect vascular responsiveness in the resistance mesenteric arteries of apoE^-/- ^mice [99]. The effect of non-pharmacological therapies on vascular dysfunction in hypercholesterolemic mice has also been tested. For example, physical exercise has been shown to prevent the progression of atherosclerotic lesions and the endothelial NO-dependent dysfunction of thoracic aortas from apoE^-/- ^mice [73,85]. This effect is likely a result of improving the sensitivity of vasorelaxations induced by vasodilating agents and shear stress and by improving the efficiency of signaling events that lead to an increase in NO bioactivity [19,73]. The above data also provide a rationale for further studies aimed at testing new preventive and corrective therapies for endothelial dysfunction in atherosclerotic disease. In this context, there is an urgent need for the development of sensitive and specific biomarkers to assess the oxidant status of patients with endothelial dysfunction.

### The endothelial dysfunction in resistance vessels

**Figure 7:**
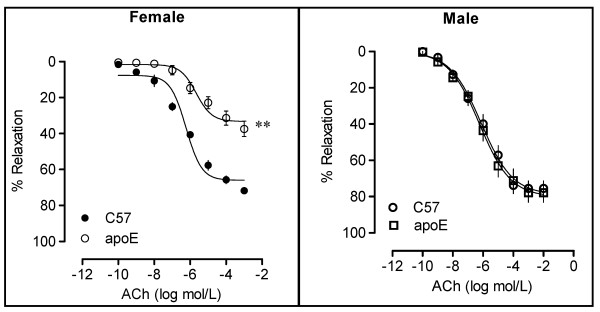
**Graphs showing impaired endothelium-dependent relaxation in response to acetylcholine (ACh) in mesenteric resistance arteries of female but not male apoE^-/- ^mice fed a regular chow diet**. The values are means ± SEM. **p < 0.01 compared with wild-type (C57) mice. Reproduced with permission from Arruda et al. [102] (right panel) and from Cola et al. [108] (left panel).

Because the vascular morphology function of the microcirculation and large vessels differ, the conclusions based on data obtained from large arteries cannot be generalized and applied to microcirculation. However, in contrast to large arteries, much less is known about the effects of hypercholesterolemia on resistance vessels, which are of physiological importance in the control of blood flow and organ perfusion. Hypercholesterolemia also impairs the endothelium NO-dependent function in the resistance arteries of patients [100], despite the fact that the resistance vessels rarely exhibit atherosclerosis. Studies have shown that young (6-19-week-old) male apoE^-/- ^mice fed a standard chow diet (hypercholesterolemia only) have a preserved endothelial NO-dependent relaxation response to ACh in cutaneous vessels [101] and in the mesenteric vascular bed [102,103]. A similar phenotype has been reported in the mesenteric arteries [20,99] and skeletal muscle resistance arterioles [16,104] in adult male (20-40-week-old) mice. Interestingly, the coronary resistance vessels from this murine model fed a regular chow diet do not exhibit impaired vasodilator responses to ACh (or to PGE_2 _mimetics), but they do exhibit impaired vasodilator response to bradykinin [105,106]. Nevertheless, when apoE^-/- ^mice are fed a Western-type diet, the ACh-induced relaxation responses in coronary arterioles and in segments of mesenteric arteries are attenuated and increased levels of ET-1 are observed [20,106,107]. As outlined for large vessels, gender can also be an important factor that influences endothelial dysfunction in resistance vessels. Indeed, as shown in Figure 7, an impaired relaxation response to ACh in resistance mesenteric arteries was reported in female [108], but not in male [102] apoE^-/- ^mice fed a standard chow diet. Reduced responses to ACh were also reported in cerebral arterioles from female apoE^-/- ^mice fed either normal or high-fat diets despite the absence of atherosclerotic lesions in those vessels [109]. In the same study, the authors also noted that although female apoE^-/- ^mice on high-fat diet had higher total PC levels and more extensive atherosclerotic lesions in the aorta than either the control or apoE^-/- ^mice on a normal diet, the impairment of the responses to ACh was similar in on both normal and high-fat diet apoE^-/- ^mice. Hypercholesterolemia, even without morphological changes in resistance vessels, is associated with endothelial dysfunction in females but is not consistently observed in males. Therefore, one must be cautious when interpreting these results because female apoE^-/- ^mice may be more susceptible to developing endothelial dysfunction, as suggested by evidence showing that females are more prone to developing atherosclerosis than males [5,21,24,36].

### The role of NO, ROS and endothelin in endothelial dysfunction of resistance vessels

It has been proposed that in resistance vessels in mice, the predominant agonist-induced endothelium-dependent vasodilation is not mediated by NO, PGI_2 _or sGC but by H_2_O_2 _or an EDHF-like principle agent [54,110]. Accordingly, in the cerebral arterioles of hypercholesterolemic female mice, endothelium-dependent dilator responses are improved by treatment with the cell permeable SOD mimetic tempol, a superoxide scavenger, and apocynin, an inhibitor of NADPH oxidase [109], suggesting that hypercholesterolemia may modulate cerebral arteriolar dysfunction, at least in part, via NADPH oxidase-derived •O_2_^-^. Likewise, coronary resistance vessels from apoE^-/- ^mice demonstrate a preserved function of the PGI_2 _system; however, NADPH induced •O_2_^- ^formation was enhanced in cardiac extracts from hearts, and the vasodilator response to bradykinin was practically abolished by an SOD mimetic [105] indicating that the endothelial dysfunction in these vessels is likely mediated by the inactivation of bioavailable NO by •O_2_^-^. Moreover, others have shown that in apoE^-/- ^mice fed a Western-type diet, the impairment of the vasodilator response of coronary arterioles to ACh was partially restored by the NADPH oxidase inhibitor apocynin [107]. These findings indicate that the endothelial dysfunction in coronary resistance vessels is not due to a reduced eNOS expression, but is most likely a result of the inactivation of bioavailable NO by •O_2_^-^. However, in mesenteric arteries from male apoE^-/- ^mice, the impaired relaxation response to ACh was partially inhibited by L-NAME but the remaining portion of the response was not attenuated further by indomethacin. However, treatment with the ET antagonist darusentan restored normal endothelial function [20], suggesting that endothelial NO, but not endothelium-derived prostanoids, mediates a portion of the relaxation response to ACh and this observation highlights the contribution of ET-1 to endothelial dysfunction. Thus, in the apoE^-/- ^mouse, the major contribution to the endothelial dysfunction in resistance vessels appears to be from the increase in NADPH oxidase-derived •O_2_^- ^and ET-1.

### The balance between vasoconstrictor and vasodilator responsiveness in conducting and resistance vessels

**Figure 8:**
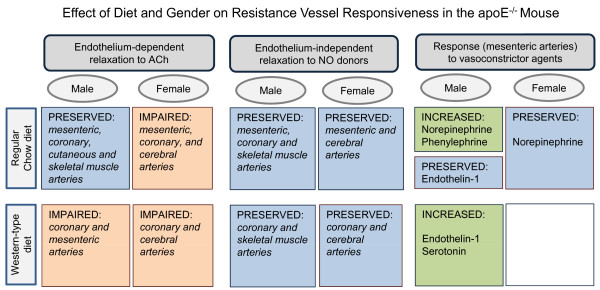
**Diagram summarizing the effect of diet and gender on endothelium-dependent relaxation in response to acetylcholine (ACh) and endothelium-independent relaxation in response to nitric oxide (NO) donors, and the response to vasoconstrictor agents in resistance arteries of apoE^-/- ^mice, based on majority of studies reviewed**.

Local vascular control depends on the balance between dilators and constrictors; thus vascular dysfunction, a term that is most often used to describe the impairment of endothelium NO-dependent vasodilatation, also involves changes in the vasoconstrictor response to endogenous and exogenous agents (see diagram in Figure 8). For local vascular control, the major opposition to vasodilator substances is ET-1 and this is in addition to Ang II from the renin-angiotensin system, which exists in the vascular endothelium [57]. In other species, hyperlipidemia has been associated with altered vasoconstrictor responsiveness in large arteries [111,112]. These studies demonstrated that vasoconstrictor responses to NE are increased in hypercholesterolemic rabbits and monkeys prior to their development of atherosclerosis. Arruda et al. [102] and Pereira et al. [103] showed that in mesenteric resistance arteries from male apoE^-/- ^mice fed a normal chow diet, vasoconstriction in response to norepinephrine was exacerbated despite a preserved vasodilation response to ACh, which could be attributed to increased oxidative stress, as indicated by the finding that apocynin (a NADPH oxidase inhibitor) normalizes the increased vasoconstriction induced by the α_1_-adrenoceptor agonist phenylephrine (PE) in hypercholesterolemic mice [86]. On the one hand, when fed a standard diet, apoE^-/- ^mice lesion-free thoracic aortas, which exhibit normal ACh-induced relaxations, exhibit an exacerbated sensitivity to PE, which is attributable to an attenuated basal bioavailability of NO [63]. On the other hand, young apoE^-/- ^mice fed a Western-type diet exhibit a normal aorta contractile response to PE but an augmented vasoconstrictor response to ET-1 [97]. However, when apoE^-/- ^mice were fed a Western-type diet, which resulted in attenuation of the ACh-induced relaxation response in segments of the mesenteric arteries, increased levels of ET-1 and enhanced contractions in response to ET-1 and serotonin were observed [20]. In addition, the authors showed that treatment with the ET antagonist darusentan normalized the endothelium-dependent relaxation responses. Thus, the vascular dysfunction characterized by hyperresponsiveness of conducting and resistance vessels to α_1_-adrenoceptor agonists appears to be due to increased oxidative stress. The studies outlined above also suggest a remarkable contribution of the endogenous ET-1 peptide to endothelial dysfunction, primarily in resistance arteries.

### The association of DNA damage with endothelial dysfunction and its treatment with new therapies

**Figure 9:**
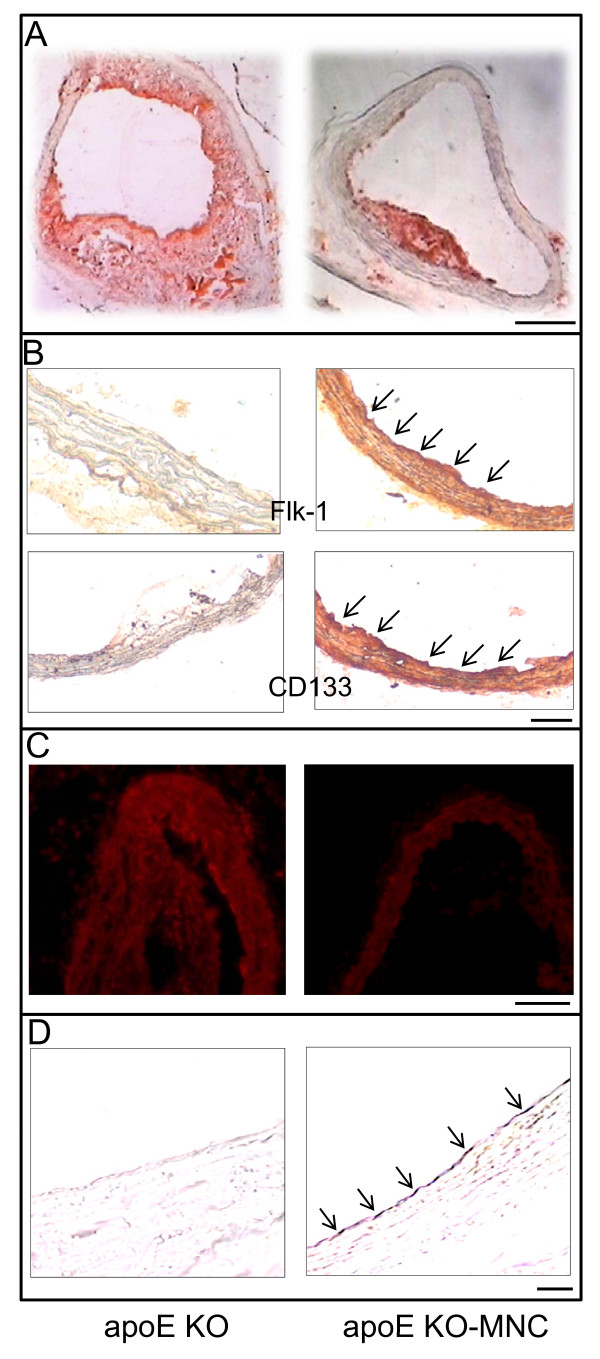
**Representative photomicrographs showing the effect of mononuclear cell (MNC) therapy on (A) lipid deposition (Oil-Red-O staining), (B) homing of endothelial progenitor cells (stained with markers Flk-1 for vascular endothelial growth factor receptor and CD133 for hematopoietic stem cell antigen), (C) superoxide anion production (dihydroethidium staining), and (D) eNOS production (DAB staining) in the aortas of female apoE KO mice**. Bar: 100 μm (A) and 50 μm (B, C and D). Reproduced with permission from Porto et al. [124].

A number of studies support the concept that there is a link between the ROS-induced oxidative damage to DNA in atherosclerosis and the overexpression of poly(ADP-ribose) polymerase (PARP) [113-116]. However, studies of the effects of acute and chronic PARP inhibition on the ability of the aorta to relax in response to ACh in apoE^-/- ^mice have produced conflicting conclusions. Pacher et al. [70] reported that the activation of PARP is associated with hypertension and aging, but not with atherosclerosis. In contrast, others [71,113] have shown that functional alterations in the endothelium, at least in the apoE^-/- ^mouse, are dependent on the activation of PARP in endothelial cells. Excessive oxidative stress and DNA damage have also been associated with vascular senescence, which was originally described as the limited ability of a cell to divide, and this could contribute to the pathogenesis of age-associated vascular disorders [117,118]. However, recent studies in our laboratory showed that, at least in the aorta, vascular senescence is present in atherosclerotic aged apoE^-/- ^mice but not in non-atherosclerotic aged wild-type C57mice [119], indicating that the occurrence of vascular senescence in aging needs to be associated with a vascular disease. The findings that vascular endothelial cells with senescence-associated phenotypes are present in human atherosclerotic lesions [120] and in atherosclerotic aged apoE^-/- ^mice [119] leads us to hypothesize that the senescence of vascular endothelial cells may be involved in endothelial dysfunction. The first evidence of this arises from the observation that the induction of senescence in human aortic endothelial cells by inhibiting telomere function results in decrease in eNOS activity [120,121]. Thus, the apoE^-/- ^mouse model offers an opportunity to examine and understand the interaction of vascular endothelial dysfunction and senescence that is associated with the pathogenesis of atherosclerosis. However, there is consistent evidence [122], that the atherosclerotic process initiated by endothelial death in specific areas results in their subsequent replacement by endothelial progenitor cells and that cellular repair by progenitor cells of ongoing vascular injury may be important for vascular integrity and function. Indeed, treatment with spleen-derived mononuclear cells increases vascular NOS activity and restores endothelium-dependent relaxation in the aorta of apoE^-/- ^mice [123]. This effect could be explained by our recent observation [124] that mononuclear cell therapy in apoE^-/- ^mice results in the homing of endothelial progenitor cells, a decrease in oxidative stress and an upregulation of eNOS expression (Figure 9). Therefore, cell therapy is a promising tool for the restoration of endothelial function and prevention of atherosclerosis development. Interestingly, a recent report [125] shows that sildenafil, a PDE5 inhibitor that increase NO-driven cGMP levels (see scheme in Figure 3), increases the number of bone marrow-derived endothelial progenitor cells and improves ischemia-induced neovascularization in hypercholesterolemic apoE^-/- ^mice. The results of this pharmacological therapy [125] and other cell therapy [123,124] studies in the apoE^-/- ^mouse supports ongoing studies in our laboratory, in which both therapies are associated with the purpose to obtain a better improvement of endothelial function in this murine model of hypercholesterolemia and atherosclerosis.

## Conclusion

**Figure 10:**
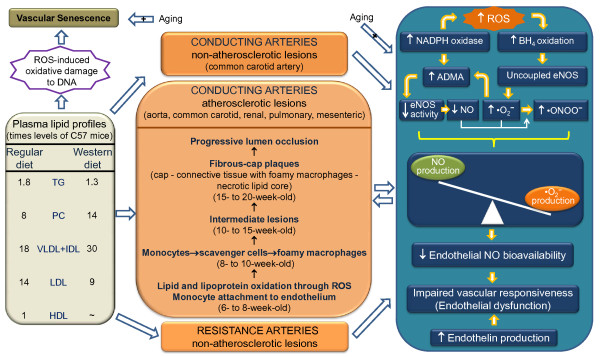
**Scheme summarizing the main lipid abnormalities and their consequence to atherosclerosis and to abnormal vascular responsiveness mediated through a NO/ROS imbalance in the apoE^-/- ^mouse**. Abbreviations: ADMA, asymmetric dimethyl-L-arginine; BH_4_, tetrahydrobiopterin; eNOS, endothelial nitric oxide synthase; HDL, High-density lipoprotein; IDL, intermediate-density lipoprotein; LDL, low-density lipoprotein; NADPH, nicotinamide adenine dinucleotide phosphate; NO, nitric oxide; •O_2_^-^, superoxide anion; •ONOO^-^, peroxynitrite; PC, total plasma cholesterol; ROS, reactive oxygen species; TG, triglycerides; VLDL, very-low-density lipoproteins.

Since its creation two decades ago, the apoE^-/- ^mouse, which spontaneously develops hypercholesterolemia and vascular atherosclerotic lesions even when fed a regular chow diet, has provided us with excellent opportunities for investigating the role of apoE in lipid metabolism and to the disease process of atherosclerosis. In this review, we show that the influence of gender on the development of atherosclerotic lesions is controversial, but evidence of the detrimental effects of estrogen on atherosclerosis has emerged during the last decade. Thus, the influence of female gender, associated with age and type of diet, on atherosclerotic lesions and endothelial dysfunction in the apoE^-/- ^mouse are expected to be subject of intense research. Several studies provide unquestionable evidence of endothelial dysfunction in conducting and resistance vessels in the apoE^-/- ^mouse. Most of these studies show that at the early stages of the disease, aortas retain their normal endothelial function but at the later stages of the disease and when mice are fed a Western-type diet, conducting vessels exhibit focal (plaque related) impairment of endothelial NO-mediated dilation. The dysfunction of the large vessels in this murine model is mainly a result of the reduced bioavailability of NO due to decreased eNOS activity and/or the chemical inactivation of NO by •O_2_^-^, and the activation of the ET-1 system. In resistance vessels, the major contribution to the endothelial dysfunction appears to be an increase in NADPH oxidase-derived •O_2_^-^, EDHF and ET-1. Despite the lack of studies focusing specifically on the influence of gender on endothelial dysfunction in apoE^-/- ^mice, there is some evidence that endothelial dysfunction in both conducting and resistance vessels is influenced by gender, aging and a Western-type diet. Scheme in Figure 10 summarizes the main lipid abnormalities and their consequence to atherosclerosis and to abnormal vascular responsiveness mediated through a NO/ROS imbalance in the apoE^-/- ^mouse. From a clinical perspective, as the mechanisms involved in the vascular reduction in NO bioavailability and the excessive production of ROS become clear, more specific therapies to prevent this defect will be developed that will ultimately lead to the correction of endothelial dysfunction. In particular, studies investigating the use of cell therapy to restore vascular function constitute a promising avenue of research.

## Competing interests

The authors declare that they have no competing interests.

## Authors' contributions

ECV, VAP, TMCP and SSM contributed equally to the conception and preparation of this review. All authors read and approved the final manuscript.
